# AYA ‘Can-Sleep’ programme: protocol for a stepped-care, cognitive behavioural therapy-based approach to the management of sleep difficulties in adolescents and young adults with cancer

**DOI:** 10.1186/s40814-022-01128-7

**Published:** 2022-07-28

**Authors:** Emma Vaughan, Maria Ftanou, Jeremy Lewin, Andrew Murnane, Ilana Berger, Joshua F. Wiley, Martha Hickey, Dani Bullen, Michael Jefford, Jeremy Goldin, Jeremy Stonehouse, Kate Thompson

**Affiliations:** 1 Psychosocial Oncology, Peter MacCallum Cancer Centre, Melbourne, Victoria Australia; 2 ONTrac at Peter Mac Victorian Adolescent and Young Adult Cancer Service, Peter MacCallum Cancer Centre, Melbourne, Victoria Australia; 3 University of Melbourne, Melbourne, Victoria Australia; 4 Sir Peter MacCallum Department of Oncology, Peter MacCallum Cancer Centre, Melbourne, Victoria Australia; 5 Behavioural Medicine Unit, Monash University, Melbourne, Victoria Australia; 6 Women’s Gynaecology Research Centre, Royal Women’s Hospital, Melbourne, Victoria Australia; 7 Australian Cancer Survivorship Centre, Peter MacCallum Cancer Centre, Melbourne, Victoria Australia; 8 Health Services Research & Implementation Science, Peter MacCallum Cancer Centre, Melbourne, Victoria Australia; 9 Respiratory Medicine and Sleep Disorders, Royal Melbourne Hospital, Melbourne, Victoria Australia

**Keywords:** Adolescent, Young adult, Cancer, Sleep, Insomnia, Stepped care, Cognitive behavioural therapy

## Abstract

**Background:**

Sleep problems are reported in up to 50% of adolescents and young adults (AYA) with cancer. Cognitive behavioural therapy for insomnia (CBTi) is considered the gold-standard treatment. In the AYA population, CBTi is associated with improvements in insomnia, daytime sleepiness, fatigue and quality of life. In adults, stepped-care interventions can improve accessibility to CBTi. This study aims to evaluate the acceptability and feasibility of a stepped-care CBTi programme in AYA with cancer.

**Methods and analysis:**

AYA (target *N* = 80) aged 16–25 with a diagnosis of cancer will be screened using the Insomnia Severity Index (ISI) and Epworth Sleepiness Scale (ESS). When sleep difficulties are identified by the ISI and/or ESS, they will be screened for obstructive sleep apnoea and restless leg syndrome and referred to a sleep service if indicated. The remainder with sleep difficulties will be offered a stepped-care sleep programme including CBT self-management and coaching (first step). Participants will then be rescreened at 5 weeks, and those with ongoing sleep difficulties will be offered individualised CBT (second step). Recruitment and retention rates, adherence to intervention and time taken to deliver screening and intervention will be collected to assess the feasibility of the programme. AYA and clinicians will complete evaluation surveys to assess the acceptability of the AYA Can-Sleep programme.

**Discussion:**

We seek to contribute to the evidence base regarding screening and treatment of sleep difficulties in the AYA population by implementing the AYA Can-Sleep programme and determining its feasibility and acceptability as an approach to care in an Adolescent & Young Adult Cancer Service.

## Background

Each year in Australia, over 1200 adolescents and young adults (AYA) aged between 15 and 25 years are diagnosed with cancer [1]. At this life stage, a cancer diagnosis comes at a critical time of growth and development with far-reaching consequences for both physical and psychosocial health [2], extending into the post-treatment survivorship phase [3, 4]. Sleep problems are among the most commonly reported consequences of cancer treatment, with up to 50% of AYA experiencing sleep disturbance and insomnia before, during and after treatment [5–7].

The most commonly reported sleep problems for AYA include problems initiating sleep, remaining asleep, excessive daytime sleepiness, fragmented sleep and excessive napping [7]. Sleep problems in AYA have been associated with emotional regulation difficulties, deficits in social skills and cognition, school and work challenges, and higher rates of depression, anxiety and posttraumatic stress disorder (PTSD) [8]. Developmentally, AYA are at increased risk biologically and behaviourally for sleep difficulties [9, 10]. Changes in circadian and hormonal processes together with external factors such as social commitments, evening technology use, high homework load or evening work lead to later bedtimes and shorter sleep duration [11, 12]. When an AYA is diagnosed with cancer, it is common for them to experience further disruptions to sleep due to factors such as frequent hospitalisations, the physical symptoms of cancer, medication and treatment side effects [6]. Following treatment, AYA are faced with many additional stressors, including ongoing physical symptoms, stress about returning to school and/or work, anxiety about a recurrence of their cancer, and ongoing medical demands [6].

Cognitive behavioural therapy (CBT) is a psychological intervention that aims to change thoughts, behaviours and emotions that can interfere or exacerbate sleep problems [13]. CBT for insomnia is delivered as a multicomponent intervention and may include sleep restriction, stimulus control, sleep hygiene, cognitive restructuring and relaxation training [14]. Whilst research has demonstrated its effectiveness in many patient populations, including adults with cancer [15], few studies have focused on its effectiveness on the AYA population. In a study with 12 AYA with cancer, Zhou et al. (2017) piloted a CBT for insomnia programme, which consisted of three individual sessions (approximately 14 days apart) with a clinical psychologist. Sleep variables were found to improve immediately post intervention (at session 3) and were sustained at 2-month follow-up [16]. In a more recent study by Zhou et al. (2020), a CBT programme called Sleep Healthy using the Internet (SHUTi) was delivered to AYA with cancer [17]. Consisting of six sessions, SHUTi is based on the fundamental strategies from CBT for insomnia and is delivered via an automated Internet programme. At the completion of SHUTi, AYA experienced improvements in several domains including daytime sleepiness, fatigue, and quality of life. These improvements were maintained up to 2 months post intervention. The SHUT-I programme however now requires a subscription (approximately US $200 per person) which may not be feasible for AYA or cancer services.

Traditional CBT is delivered through 50–60 min face-to-face sessions, with one psychologist/counsellor to one person [15]. This model is expensive and often does not fit the needs of AYA who are also juggling work, school and other social commitments. Another significant barrier in the treatment of sleep difficulties is the shortage of experts trained in CBT both in the community and in cancer centres across the world [18]. An option to improve accessibility and feasibility is self-management CBT resources. A meta-analysis of randomised controlled trials found that self-management CBT resources are an efficacious and acceptable alternative for the treatment of insomnia, especially when telephone consultation was included to encourage participation and enhance adherence [19]. Self-management CBT resources have been found to be effective in both adults with cancer [20] and AYA with cancer [17].

Whilst self-management CBT resources are an efficacious alternative, a meta-analysis indicated that sleep improvements were consistently of a lower magnitude than those from face-to-face CBT and were not a substitute for professionally administered treatment [21]. A meta-analysis of randomised controlled trials recommended the use of self-management CBT as an entry level of a stepped-care model for insomnia [19]. Stepped-care models allow for more rapid access to mental health services in a wide range of settings [22]. They are based on the premise that interventions should vary in type and intensity, especially in healthcare systems with limited resources [23]. In a stepped-care model, an entry level treatment should be the most readily accessible, cost effective, least restrictive and able to provide signficant health gain, such as self-management CBT [24]. Treatment intensity can then be stepped up if the entry level treatment is not providing significant health gain [24, 25]. With a stepped-care model to manage insomnia, the more intensive treatments (face-to-face sessions) are reserved for people who do not benefit from less intensive self-management CBT; as a result, treatment costs are reduced, and resource allocation is maximised [26].

Stepped-care interventions developed for adults with cancer have been shown to improve the accessibility to CBT for treatment of sleep difficulties [27]. There have been no published trials of stepped care for sleep difficulties in the AYA population. This study aims to evaluate the first known stepped-care programme to treat sleep difficulties in AYA with cancer.

## Methods and analysis

### Design

This study design is a prospective, single-arm study to evaluate feasibility and preliminary efficacy of an adapted stepped-care sleep intervention for AYA with sleep difficulties and cancer.

### Study setting

Outpatient cancer clinics across two specialist metropolitan hospitals in Melbourne, Australia.

### Sample size

As this is a feasibility study, a power calculation to determine sample size was not undertaken. The target sample for screening is 80 AYA with cancer across a 15-week period. This is based on expected numbers of people seen in a large tertiary cancer centre and is in line with other sleep studies in the AYA population [6, 28]. This number is also consistent with recommendations for pilot and feasibility studies [29–31] and will provide data on the acceptability and feasibility of the AYA Can-Sleep programme.

### Participants

The study includes two groups: AYA with cancer and healthcare professionals delivering cancer care to the AYA population.

### Participant inclusion criteria

#### AYA

To be eligible to participate in this study, the following criteria must be met: (i) aged between 16 and 25 years of age, (ii) have a histologically confirmed diagnosis of cancer, (iii) able to give informed consent (i.e. no psychiatric/cognitive condition that would impact informed consent, as based on clinical judgement) and (iv) able to read and write in English.

#### Healthcare professionals

To be eligible to participate in this study, healthcare professionals must be working in the outpatient cancer clinics involved in the study. These healthcare professionals may include oncologists (adult and paediatric), nurses, psychosocial oncology clinicians and allied health staff.

### Participant recruitment and consent

#### AYA

AYA will be recruited from outpatient clinics at the Peter MacCallum Cancer Centre and the Royal Women’s Hospital, Melbourne. Hospital clinic lists will be screened by the research team. AYA who meet the inclusion criteria will be approached by either a psychologist or a member of the clinical team, verbally informed of the project and invited to take part in the AYA Can-Sleep programme and its evaluation. Interested AYA will be provided with verbal and written information on the project, including study requirements and participant information and consent form (PICF). Written informed consent will be obtained in clinic by participants who agree to participate. Alternatively, for potential participants who wish to consider their involvement, an online link to the PICF will be sent via REDCap. AYA who do not return their PICF within 2 weeks of receiving it will be followed up with one phone call/voicemail and one email reminder. Those who still do not return the PICF will be considered to have declined involvement and have no further contact from the project team.

AYA who decline to participate in the study will be asked if they consent to their reasons for declining, patient information (age, sex, diagnosis, treatment received) and site they were approached at being recorded. This will allow for differences between consenters and decliners to be examined.

#### Healthcare professionals

Clinicians who have participated in the AYA Can-Sleep programme will be sent a link to the clinician engagement survey at the end of the evaluation period. Consent will be implied when clinicians return completed surveys.

### Study measures

Study measures and the timing of assessments are summarised in Table 1.

**Table 1:** Schedule of enrolment and assessments

	Item/time	Enrolment	Rescreen	Completion
		*t*_0_	*t*_1_	*t*_2_
**Screening**				
Insomnia Severity Index	7	X	X	X
≥ 8 indicates symptomatic				
≥ 3-point change is clinically meaningful				
Epworth Sleepiness Scale	8	X	X	X
≥ 10 indicates symptomatic				
STOP-BANG	8	X		
≥ 5 are classified as high risk of moderate to severe OSA				
Restless Legs Syndrome Scale	5	X		
−5/5 at risk of restless leg syndrome				
**Feasibility**				
Proportion of people approached who consent	-	X	X	X
> 50% indicates programme is feasible				
Dropout rate	-	X	X	X
> 40% indicates programme is feasible				
**Acceptability** — *quantitative and qualitative data*				
Participant experiences survey — screening questionnaires	5	X		
Participant experiences survey — post follow-up care	10		X	X
Clinician engagement survey	14			X
Other variables				
Demographic and cancer information	4	X		
Administrative and fidelity data		X	X	X

X, measure administered at that time point

### Demographics and medical history

Following consent to the project, demographic and clinical characteristics will be collected from the participant’s medical record, including age, gender, diagnosis and treatment received.

### Assessment for sleep difficulties

#### *The Insomnia Severity Index (ISI)* [32]

The ISI is a 7-item self-report measure that assesses the severity of sleep difficulties. It assesses problems falling asleep, maintaining sleep and early morning awakening. It also assesses satisfaction with current sleep, noticeability of sleep problems to others, worry about sleep and the interference of sleep problems with daily functioning. The items on the ISI are rated on a Likert scale and summed to obtain a total score, ranging from 0 to 28. Higher scores represent more significant insomnia difficulties. A cutoff of ≥ 8 indicates sleep difficulties and will be used as the cutoff for this study [32–34]. The ISI has good internal consistency reliability (alpha = 0.83), test-retest reliability of 0.79 and has been validated in AYA with cancer [33].

#### *The Epworth Sleepiness Scale (ESS)* [35]

The ESS is an 8-item self-report measure that asks participants to rate the probability of falling asleep on a scale of increasing probability from 0 to 3 for different situations that most people engage in during their daily lives including “watching TV” or “sitting and reading.” Scores of ≥ 11 will be interpreted as clinically significant, as scores from 11 to 24 represent increasing levels of excessive daytime sleepiness. The ESS has good internal consistency reliability (alpha = 0.89) and a 2-week test-retest reliability of 0.82 in both the adult and AYA populations [36].

#### *STOP-BANG* [37]

STOP-BANG is an 8-item self-report questionnaire that assesses the presence of risk factors for obstructive sleep apnoea (OSA). Items are dichotomous (yes/no), and total scores range from 0 to 8, with higher scores indicating higher risk of OSA. Scores of 5 to 8 are classified as high risk of moderate to severe OSA. STOP-BANG has demonstrated adequate sensitivity of up to 96% to detect OSA [38].

### Restless Leg Screening Scale (RLSS)

The RLSS is a purpose built self-report measure comprising of 5 dichotomous items (yes/no) which reflect the diagnostic criteria for restless leg syndrome in DSM-V. Where all five items are rated as ‘yes’, the AYA is considered at risk of restless leg syndrome.

#### Acceptability and feasibility

Three purposely designed experience surveys will be administered to assess the acceptability of screening and interventions from the perspective of both AYA and clinicians. These surveys have been developed using the reach, effectiveness, adoption, implementation and maintenance (RE-AIM) framework to evaluate the acceptability, feasibility and effectiveness of the AYA Can-Sleep programme from both young people and clinicians [39, 40].

### Participants experience survey — screening questionnaires

This survey is a purpose built 6-item measure designed to assess the following: (1) how well the reasons and procedure for completing the AYA Can-Sleep programme screening questionnaires were explained, (2) how easy the screening questionnaires were to understand and complete and (3) whether the completion time was acceptable or too long. Survey completion time is no more than 5 min, and participants will complete this survey only once after initial screening.

### Participant experiences survey — post follow-up care

This 10-item survey has been designed to the following: (1) identify the most useful/helpful and least useful/helpful aspects of the follow-up care they received, (2) to determine what barriers have prevented seeking help for sleep problems in the past and (3) to assess whether AYA experienced any subjective changes in their sleep following the intervention they received. Survey completion time is approximately 10 min, and participants will be asked to complete this survey within 2 weeks of completing the follow-up care they receive.

### Clinician engagement survey

This 14-item survey aims to elicit information about positive and negative experiences clinicians have with the AYA Can-Sleep programme including the following: (1) the impact the AYA Can-Sleep programme has had on AYA and patient care, (2) the impact the AYA Can-Sleep programme has on their service, (3) what assistance they provided AYA with sleep difficulties prior to the AYA Can-Sleep programme and (4) how they found the process of referring patients into AYA Can-Sleep programme. This information will help to assess the acceptability of the programme and assist in the improvements required to integrate it into routine practice. Survey completion time is approximately 20 min.

### Operational data

Clinician time taken to deliver screening and intervention will be collected on a project-specific case report form. Referral rates, participant uptake of follow-up care, number of weeks completed of intervention received, uptake of evaluation participation of both AYAs and clinicians and those that refuse the intervention despite meeting eligibility criteria (including reasons for declining participation) will also be collected to assess feasibility of the program.

## Study procedures

### Initial screening and referral

Over a planned 15-week period, all participating AYA will be screened for insomnia using the ISI and ESS. Participants with scores of < 8 on ISI and score of < 11 ESS will receive no follow-up or treatment. Participants who are identified as having sleep difficulties (scores of ISI ≥ 8 and/or ESS ≥ 11) will then be asked to complete questionnaires for obstructive sleep apnoea (STOP-BANG) and restless legs syndrome (RLSS).

Participants who score high on the STOP-BANG (≥ 5) or the RLSS (i.e. yes to all questions) will be referred to the Department of Respiratory and Sleep Medicine at the Royal Melbourne Hospital, Melbourne, Australia, for further assessment. Participants who score below cutoffs on both the STOP-BANG and RLSS will be offered the AYA Can-Sleep stepped-care programme.

### Stepped-care interventions

**Fig. 1:**
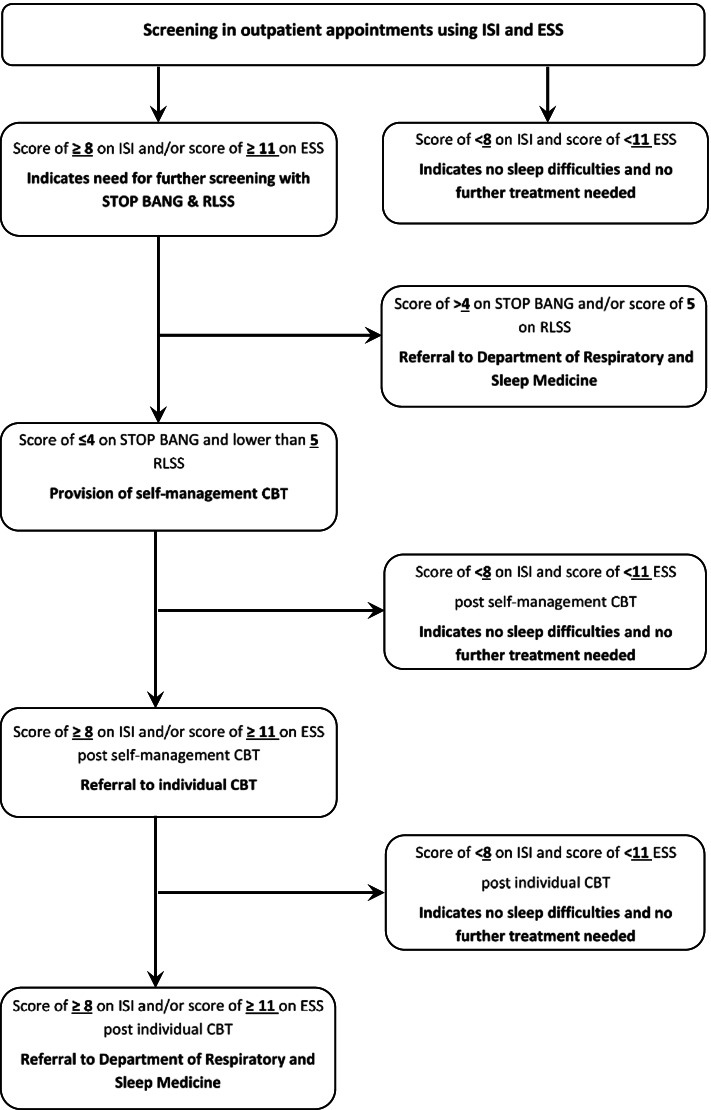
Referral pathways for the ‘AYA Can-Sleep’ stepped-care programme. ISI, Insomnia Severity Index; ESS, Epworth Sleepiness Scale; STOP-BANG, obstructive sleep apnoea measure; RLSS, Restless Legs Screening Scale; CBT, Cognitive Behaviour Therapy

The stepped-care interventions are described in detail below, and Fig. 1 demonstrates how participants will be referred in to each part of the AYA Can-Sleep programme.

### CBT self-management resource (CBT-SM)

#### Design of CBT-SM

The project team partnered with the Victorian and Tasmanian Youth Cancer Action Board (YCAB) to adapt the Can-Sleep resource titled: “Can-Sleep Making night-time sleep problems go away” [41] for the AYA population. The Can-Sleep resource, developed initially for the adult cancer survivor population, by adults, was adapted to ensure it was more engaging, satisfying and acceptable to young people. YCAB is a 12-member board of young people who have had a diagnosis of cancer between the ages of 15 and 25 years. They are broadly representative of the diversity of young people and have received treatment across the paediatric/adult, metro/regional and public/private healthcare sectors and have a strong track record in the co-design and development of resources for young people in the area.

A face-to-face workshop was conducted with YCAB, where AYA were provided with prereading materials including a copy of the current Can-Sleep resource and questions/reflections to prepare for workshop discussions. The workshop commenced with revisiting the rationale behind the development of targeted sleep intervention for AYA cancer survivors. It then explored participant’s experiences with sleep-related challenges both during and following treatment completion. This was followed by a brainstorming exercise to gather suggestions regarding content, style and design of the resource. The resource has now been redesigned and rewritten based on feedback from AYA and has been endorsed by the YCAB.

### Step 1: CBT self-management resource (CBT-SM)

Participants referred to step 1 will be provided with the CBT self-management resource (CBT-SM). The CBT-SM resource includes psychoeducational material and strategies on key cognitive behavioural techniques to manage sleep difficulties. This includes the following: practical advice on how to establish good sleep hygiene habits and use relaxation techniques to promote sleep, strategies to manage common worries and cognitions that interfere with sleep and strategies to manage common cancer treatment side effects that interfere with sleep including pain and unpleasant night-time sensations including nausea, hot flushes, rash and symptoms of peripheral neuropathy.

A psychologist or member of the clinical team trained in administering the resource will provide the CBT-SM resource to the AYA and provide instructions for its use. Approximately, 3 weeks post receiving the CBT-SM resource, AYA will be contacted (via text, email or phone) by a psychologist to answer any questions the AYA might have and resolve any problems that may have arisen.

Five weeks post receiving the CBT-SM, participants will be rescreened using the ISI and ESS. For participants with scores of ISI < 8 and ESS < 11 following CBT-SM, it will be assumed that their sleep difficulties have improved, and they will receive no further treatment. If participants have ongoing sleep difficulties (i.e. scores of ≥ 8 on the ISI and/or ≥ 11 on the ESS), they will be referred for individual CBT. If the participant cannot attend individual CBT, they will be referred back to community support services and/or their GP for follow-up.

### Individual CBT

Participants referred to individual CBT will be invited to attend 4 structured individual CBT sessions. The individual CBT sessions will be of approximately 50–60-min duration, with a psychologist and will be conducted either face to face or via telehealth depending on the participant’s preference. The individual CBT sessions will include all the information provided in the CBT-SM resource and provide an opportunity to identify and address barriers to change. The content of the individual CBT sessions has been modelled on CBT insomnia programmes that have been efficacious in the adult cancer population [42] and the AYA cancer population [16]. The session structure is outlined in Table 2 below.

**Table 2:** Session structure of individual CBT

Session	Content
1	• Conduct an evaluation of sleep history • Further education around sleep hygiene • Introduce the rationale for sleep restriction and stimulus control and address any potential barriers • Discuss cancer-related late effects and medications that impact sleep function • Provide education on completion of a sleep diary
2	• Review of sleep diary • Provide education on the calculation of sleep efficiency • Schedule sleep-wake schedule based on sleep diary • Introduce arousal and how this affects sleep • Discuss counter arousal methods
3	• Review of sleep diary • Discuss sleep expansion • Explore participant’s beliefs about sleep • Identify and address cognitive factors that impact adherence to ‘Can-Sleep’ programme and sleep function
4	• Review progress • Discuss sleep-related cognitive arousal • Review of goals • Create a relapse prevention plan (if appropriate) • Discuss follow-up options

The intervention will address symptoms that are specific to AYA with cancer (e.g. common side effects from cancer treatment that can effect sleep function). Sleep diaries will be completed throughout individual therapy, so that sleep restriction recommendations can be tailored for each participant.

In the week after the fourth session, participants will be rescreened using the insomnia measures. Participants with ongoing sleep difficulties (score ≥ 8 on the ISI) will have a treatment planning discussion of further needs and treatment options, which may include referrals to the Department of Respiratory and Sleep Medicine, a psychiatrist, further individual therapy, peer supports or community services.

### Data analysis

Demographic, acceptability and feasibility data and survey responses will be analysed descriptively (means/SD or frequency/percentage as appropriate). The impact of the programme will be evaluated on the basis of the number of AYA who report clinically meaningful changes (improvements) in sleep quality as measured by changes in scores of insomnia measures collected at screening, post the CBT self-management resource and post individual CBT. Based on a previous study in cancer [43], clinically meaningful changes for the ISI were defined at 3 or more; a change of 3 represents approximately half a standard deviation on the ISI or about half a “category” shift based on common cutoffs. Data will be managed through REDCap, and quantitative data will be analysed using SPSS.

Qualitative responses will be analysed using thematic analysis focusing on understanding participants’ experiences within and the different stepped interventions. To analyse free-text content, analysis will be undertaken. Data from free-text responses will be coded to encapsulate the key idea of the response. Once coded, the data will be sorted to examine the frequency of responses and the content of the responses.

### Ethics and dissemination

This study, protocol and all measures, including the informed consent document, have been approved by Human Research Ethics Committee (HREC) of the Peter MacCallum Cancer Centre (HREC reference number: HREC/74976/PMCC—2021; project number 21/93L). This project will be conducted according to the NHMRC National Statement on Ethical Conduct in Human Research (2007 and updates) [44] and the World Medical Association of Helsinki (2013 and updates) [45]. The trial is registered to the Australian New Zealand Clinical Trials Registry (ANZCTR) (ACTRN: ACTRN12622000792729).

### Informed consent

AYA will be assured that participation is entirely voluntary, and that they may stop their involvement in the evaluation project at any time. It will be explained that their decision will not impact on their care or relationship with the hospital.

### Data storage and privacy issues

A unique study identification number system will be used for data collected for this project linking AYA’s personal identifying information (e.g. names, URNs) with their corresponding study identification number (e.g. PT01/PT02) Data will be collected and managed in REDCap (Research Electronic Data Capture) hosted at the Peter Mac. Only members of the project team will have access to this data, in accordance with the National Statement on Ethical Conduct in Human Research 2007 and the Australian Code for Responsible Conduct of Research 2018.

### Withdrawal criteria

AYA who do not continue with the AYA Can-Sleep follow-up intervention will be asked if they consent to complete follow-up measures, evaluation, and for any of their existing data to be included in analyses. If consent is not given for the latter, their data will be erased from the database at the completion of the study, and any electronic or paper records pertaining to their involvement will be destroyed except medical notes that have been committed to the electronic system.

A record of AYA who have withdrawn from the study will be recorded and maintained on the secure REDCap database until the completion of the study. This is to ensure that these AYA are not approached again by the project team. AYA will be unable to withdraw their data after the completion of the study as their data may have already been used in analyses.

### Confidentiality

It is not expected that participating in this project will pose any risks of harm to participants. If any disclosures of risks to safety (e.g. suicidal ideation) occur during any stages of the project, standard clinical processes will be followed including safety planning with the participant and when needed advising an appropriate support person such as a member of the participant’s treating team and/or a family member. The limits to confidentiality are included in the PICF.

### Dissemination

Progress and outcomes of the project will be communicated and disseminated in a number of ways. Manuscripts will be prepared for publication in peer-reviewed journals. At the completion of the program, the treatment framework and project resources will be hosted on an online platform and disseminated to other interested organisations. Project findings will also be disseminated at national and international conferences. For the duration of the project, progress against milestones will be continuously monitored and reports provided to the Victorian Government Department of Health as per the funding requirements.

## Discussion

This study aims to evaluate the first known stepped-care programme to treat sleep difficulties in AYA with cancer. This study protocol is designed to assess valuable information regarding recruitment, prevalence and severity of sleep difficulties in the AYA population, retention and completion rates and the acceptability of a stepped-care programme to treat sleep difficulties to AYA and clinicians. These findings will help to assess the feasibility and acceptability of stepped-care CBTi as a standard approach to care in an Adolescent & Young Adult Cancer Service.

## Data Availability

Not applicable
